# Maryland

**Published:** 1897-01

**Authors:** 


*Maryland.* Rabies is somewhat prevalent in Baltimore. A

number of bitten people have been sent to the Pasteur Institute in New York City, one or two of whom have died from hydrophobia.

